# Estimated pulse wave velocity, waist-to-height ratio, and risk of cardiometabolic multimorbidity: A secondary dataset analysis of the China Health and Retirement Longitudinal Study (CHARLS)

**DOI:** 10.18332/tid/216379

**Published:** 2026-03-20

**Authors:** ZhiYing Fei, LingLing Bian, ShuLin Lu, ChunQiao Wu

**Affiliations:** 1 Department of Nursing, Sir Run Run Shaw Hospital, Zhejiang University School of Medicine, Hangzhou, People’s Republic of China

**Keywords:** estimated pulse wave velocity, waist-to-height ratio, cardiometabolic multimorbidity

## Abstract

**INTRODUCTION:**

Obesity, smoking, and atherosclerosis increase the risk of developing various cardiometabolic diseases. The estimated pulse wave velocity (ePWV) is a new indicator of arterial stiffness. However, the relationship between ePWV, waist-to-height ratio (WHtR), smoking, and cardiometabolic multimorbidity (CMM) remains unclear.

**METHODS:**

The study is a secondary dataset analysis of CHARLS, which included 8414 participants from the China Health and Retirement Longitudinal Study conducted between 2011 and 2018. The ePWV was calculated using the mean blood pressure and age. Cox proportional hazards models were used to explore the relationship between ePWV, WHtR, and CMM in both smoking and non-smoking populations. Additionally, we also employed restricted cubic spline (RCS) analysis and mediation analysis to investigate the relationship between ePWV, WHtR, and CMM. A p<0.05 was considered statistically significant.

**RESULTS:**

During the 7-year follow-up period, 1545 participants (18.36%) developed new-onset CMM. The RCS model exhibited a U-shaped relationship between WHtR and CMM incidence, with a positive correlation when WHtR exceeded 0.5. Cox regression analysis revealed that ePWV and WHtR were independent predictors of CMM in both smoking and non-smoking populations. Additionally, ePWV significantly mediated the association between WHtR and CMM risk.

**CONCLUSIONS:**

Our findings indicate that ePWV and WHtR are associated with increased CMM risk. Early detection of ePWV and WHtR, combined with smoking cessation, may help identify high-risk individuals and provide a basis for future preventive research.

## INTRODUCTION

Cardiometabolic diseases (CMDs) such as stroke, diabetes, and hypertension are becoming increasingly prevalent, posing a significant burden on public health^1^. Compared to individuals with a single CMD, those with multiple face significantly higher mortality rates and shorter life expectancies^2^. Cardiometabolic multimorbidity (CMM) is defined as the coexistence of more than one of the following conditions: Type 2 diabetes, coronary heart disease, and stroke^3^. Studies have demonstrated that lifestyle factors, particularly smoking, are among the primary risk factors for the development and progression of cardiovascular diseases. Passive smokers (1/100) who are excessively exposed to tobacco smoke have approximately 30% higher risk factors for coronary artery disease (CAD) compared to active smokers (80%)^4^. However, evidence regarding emerging biomarkers (e.g. ePWV and WHtR) and CMM progression remains limited.

Obesity is a potential risk factor for CMM^5^. Generally, the relationship between obesity and CMM is assessed using body mass index (BMI) as an indicator, with some studies reporting its impact on CMM^5,6^. Fat distribution is gaining attention amid growing focus on body composition research. However, BMI cannot distinguish between fat and muscle or assess fat distribution^7^. Studies have illustrated that the waist-to-height ratio (WHtR) has superior predictive ability for cardiovascular disease than BMI^8,9^. A large-scale cross-sectional study in China demonstrated that WHtR performs significantly better in detecting CMM^10^. The effect of obesity on cardiovascular metabolic diseases (CVDs) is related to atherosclerosis^11^. The estimated pulse wave velocity (ePWV) is a non-invasive marker of arterial stiffness and has been extensively studied in cardiovascular research^12^. ePWV is calculated based on age and mean blood pressure (MBP) and can predict the CVD risk and adverse outcomes in patients. However, research on the interaction between WHtR and ePWV in CMM development is limited.

We utilized the large-scale data and prospective cohort design of the China Health and Retirement Longitudinal Study (CHARLS) from 2011 to 2018, to investigate the effects of waist-to-hip ratio (WHtR) and ankle-arm pulse wave velocity (ePWV) on CMM in both smoking and non-smoking populations, and to verify whether ePWV mediates the association between WHtR and CMM risk.

## METHODS

### Population and data sources

CHARLS was established in 2011 through multi-stage, stratified, and cluster sampling of Chinese adults aged ≥45 years, with follow-up surveys conducted every two years thereafter. This study was approved by the Beijing University Biomedical Ethics Review Committee (IRB00001052-11015), and written informed consent was obtained from all participants.

**Figure 1:**
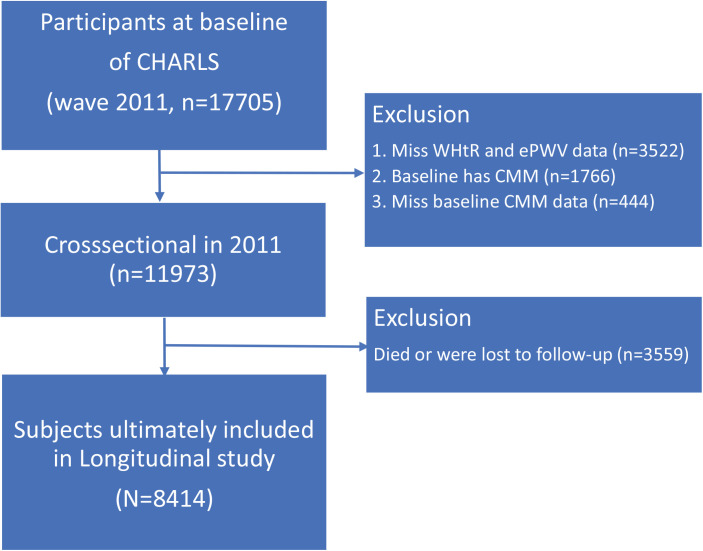
The flow chart of participants selection process

This study used a unique ID to track participants. Starting with 17705 participants at the 2011 baseline, we excluded 9291 participants for the following reasons: CMM presence at baseline (n=1766), missing CMM data (n=444), missing WHtR and ePWV data (n=3522), and missing follow-up data (n=3559). Ultimately, 8414 participants were included in this study (Figure 1).

### ePWV assessment

ePWV, a marker of arterial stiffness, is calculated using a validated formula developed by Greve et al.^13^ enabling non-invasive assessment based on age and blood pressure parameters. The calculation formula is as follows:

ePWV = 9.587 - 0.402 × age + 4.560×10^-3^ × age^2^ - 2.621×10^-5^ × age^2^ × MBP + 3.176 ×10^-3^ × age × MBP - 1.832×10^-2^ × MBP

where MBP is calculated as follows:

MBP = DBP + 0.4 × (SBP - DBP)

Blood pressure was measured using the Omron HEM-7200 device. The participants were seated with their left arm resting on a flat surface at heart level. The cuff was placed 1–2 cm above the elbow. Three consecutive readings were taken, and the average was calculated to ensure measurement reliability.

### WHtR assessment

WHtR was calculated by dividing WC by height. Height was measured using standard methods, with participants not wearing shoes. WC was measured to the nearest ±0.5 cm at the smallest circumference between the lowest ribs. The calculation formula is as follows:

WHtR = WC (cm)/height (cm)

### CMM definition

In this study, CMM was defined as having at least two CVD conditions, including diabetes, heart disease, and stroke^14^. For the CHARLS database, the diagnosis of heart disease was determined based on questionnaire responses indicating that participants had been diagnosed by a doctor with conditions such as heart failure, coronary heart disease, myocardial infarction, or other heart diseases, or were taking heart disease-related medications. Diabetes was defined as a self-reported physician’s diagnosis or the presence of any one of the following biochemical criteria: fasting blood glucose ≥7.8 mmol/L, HbA1c ≥6.0%, or random blood glucose ≥11.1 mmol/L. Additionally, individuals taking diabetes-related medications or receiving insulin injections were also classified as having diabetes. Stroke was identified based on either a participant’s report of a physician-diagnosed cerebrovascular event (such as cerebral infarction or cerebral hemorrhage) or the use of antithrombotic or other stroke-specific medications. In the clinical data, heart disease was primarily defined by discharge diagnoses including coronary heart disease, heart failure, myocardial infarction, or other heart diseases. Diabetes was determined based on discharge diagnoses of type 1 or type 2 diabetes. Stroke was identified by discharge diagnoses of cerebrovascular accidents such as cerebral infarction or cerebral hemorrhage.

### Accompanying factors

Covariates included age, sex, marital status (married and cohabiting, married but separated, and single), residence (rural, urban), education level (no junior high school education, junior high school or above), annual household expenditure, smoking habits (smoker, non-smoker), drinking habits (drinker, non-drinker), nighttime sleep duration, and BMI.

### Statistical methods

The Kolmogorov-Smirnov and Levene tests were used to assess the normality of the distribution and the equality of variances for continuous datasets. All continuous datasets were normally distributed, and continuous variables were compared using t-tests with mean ± standard deviation. Categorical variables were expressed as frequencies and percentages, and compared using the chi-squared test. We recorded the number of follow-up visits from baseline to diagnosis or 31 December 2018 (whichever occurred first). The Cox proportional hazards model was used to assess the association between ePWV, WHtR, and CMM progression in both smoking and non-smoking populations. To investigate the linear and nonlinear relationships between ePWV, WHtR, and CMM risk, both ePWV and WHtR were analyzed as continuous and categorical variables (quartiles). In the preliminary analysis, we separately established Model 1 (crude model) and Model 2 in smoking and non-smoking populations, with the latter adjusted for age, sex, residence, marital status, education level, household expenditure, alcohol consumption, and nighttime sleep duration. We also conducted a mediation analysis to quantify the influence of ePWV on WHtR and CMM. Specifically, WHtR was the predictor variable (X), ePWV served as the mediator (M), and CMM onset was the outcome variable (Y). This method has been widely used to quantify mediation effects^15^.

Additionally, we employed a four-node restricted cubic spline (RCS) function to explore potential non-linear relationships between ePWV and CMM progression, as well as between WHtR and CMM progression. We conducted several sensitivity analyses for further validation of the interaction robustness between ePWV, WHtR, and CMM onset. In sensitivity analysis 1, we excluded participants with strokes at baseline. In sensitivity analysis 2, participants with diabetes or those who had received diabetes treatment at baseline were excluded. In sensitivity analysis 3, participants with heart disease at baseline were excluded. In sensitivity analysis 4, we excluded patients who developed CMM within two years of the baseline survey to minimize latency bias.

All statistical analyses were performed using R statistical software (Version 4.3.0). Statistical significance was set at p<0.05 in each analysis.

## RESULTS

### Descriptive statistics

This study was based on a total of 8414 participants from the CHARLS survey, which was conducted from 2011 to 2018 (Table 1), with an average age of 57.6 years and 46.2% being male. During the maximum 7-year follow-up period, 1545 participants who did not have CMM at baseline (18.36% of the total) subsequently developed CMM. Table 1 presents the baseline characteristics of the study population, categorized by CMM diagnosis. Participants in the CMM group were more likely to be older, female, smokers, single, have lower household expenditure, be non-alcoholic, and have shorter nighttime sleep duration.

**Table 1:** Baseline characteristics of the longitudinal study population by CMM, CHARLS, 2011–2018 (N=8414)

*Characteristics*	*Overall (N=8414) n (%)*	*Non-CMM (N=6869) n (%)*	*CMM (N=1545) n (%)*	*p*
**Age** (years), mean (SD)	57.6 (9.2)	57.0 (9.1)	60.2 (9.1)	<0.001
**Gender**				
Female	4528 (53.8)	3635 (52.9)	893 (57.8)	0.001
Male	3886 (46.2)	3234 (47.1)	652 (42.2)	
**Residence**				
Rural	7126 (84.7)	5865 (85.4)	1261 (81.6)	<0.001
Urban	1288 (15.3)	1004 (14.6)	284 (18.4)	
**Marital status**				
Married and living with a spouse	7211 (85.7)	5922 (86.2)	1289 (83.4)	<0.001
Married but living without a spouse	350 (4.2)	299 (4.4)	51 (3.3)	
Single/divorced/windowed	853 (10.1)	648 (9.4)	205 (13.3)	
**Education level**				
Less than lower secondary education	7577 (90.1)	6185 (90.0)	1392 (90.1)	0.986
Secondary or higher	837 (9.9)	684 (10.0)	153 (9.9)	
**Smoking**				
Non-smoker	3242 (38.5)	2686 (39.1)	556 (36.0)	0.025
Smoker	5172 (61.5)	4183 (60.9)	989 (64.0)	
**Drinking**				
Non-drinker	6054 (72.0)	4879 (71.0)	1175 (76.1)	<0.001
Drinker	2360 (28.0)	1990 (29.0)	370 (23.9)	
**Sleep** (hours), mean (SD)	6.4 (1.9)	6.4 (1.9)	6.3 (1.9)	<0.001
**Household expenditure** (RMB), mean (SD)	6764.7 (8612.0)	6813.6 (8802.1)	6547.1 (7709.5)	0.272
**BMI** (kg/m^2^), mean (SD)	23.9 (32.6)	23.1 (6.6)	27.3 (74.7)	<0.001
**WHtR,** mean (SD)	0.5 (0.1)	0.5 (0.1)	0.6 (0.2)	<0.001
**ePWV** (m/s), mean (SD)	9.2 (1.8)	9.0 (1.8)	10.1 (1.8)	<0.001

CMM: cardiometabolic multimorbidity. BMI: body mass index. WHtR: waist-to-height ratio. ePWV estimated pulse wave velocity.

### Association between ePWV and CMM

*Non-smokers*

In univariate analyses, each 1-unit increase in ePWV was associated with a 28.9% increase in CMM risk (HR=1.289; 95% CI: 1.253–1.326) (Table 2). After confounding factors adjustment, the HR for CMM was 1.496 (95% CI: 1.421–1.576). EPWV was divided into quartiles, and participants in the first quartile (Q1) were used as the reference group, with an HR of 1.00. Compared with Q1, the HRs for CMM risk in the Q2, Q3, and Q4 groups were 1.994 (95% CI: 1.376–2.890), 3.193 (95% CI: 2.248–4.534), and 5.723 (95% CI: 4.088–8.013) (all p<0.001). After adjusting for confounding factors, the HR for CMM were 2.339 (95% CI: 1.604–3.412), 4.162 (95% CI: 2.871–6.033), and 9.353 (95% CI: 6.271–13.951) (all p<0.001) in subjects with Q2, Q3, and Q4 compared with those with Q1 values.

**Table 2:** Hazard ratio and 95% CI of CMM status for ePWV and WHtR, by smoking status, CHARLS, 2011–2018 (N=8414)

*Variables*	*Non-smoker (N=3242)*				*Smoker (N=5172)*			
	*Model 1*		*Model 2*		*Model 1*		*Model 2*	
	*OR (95 % CI)*	*p*	*AOR (95 % CI)*	*p*	*OR (95 % CI)*	*p*	*AOR (95 % CI)*	*p*
**ePWV** (quartiles)	1.289 (1.253–1.326)	<0.001	1.496 (1.421–1.576)	<0.001	1.335 (1.281–1.391)	<0.001	1.594 (1.487–1.709)	<0.001
Q1 (ref.)								
Q2	1.994 (1.376–2.890)	<0.001	2.339 (1.604–3.412)	<0.001	2.549 (2.001–3.245)	<0.001	2.797 (2.183–3.584)	<0.001
Q3	3.193 (2.248–4.534)	<0.001	4.162 (2.871–6.033)	<0.001	4.112 (3.274–5.166)	<0.001	4.953 (3.867–6.344)	<0.001
Q4	5.723 (4.088–8.013)	<0.001	9.353 (6.271.13.951)	<0.001	5.735 (4.596–7.158)	<0.001	8.131 (6.129–10.787)	<0.001
**WHtR** (quartiles)	2.082 (1.809–2.396)	<0.001	163.107 (61.217–434.583)	<0.001	5.860 (3.690–9.324)	<0.001	236.281 (44.672–1249.744)	<0.001
Q1 (ref.)								
Q2	1.286 (1.011–1.635)	<0.05	1.276 (1.003–1.624)	<0.05	1.434 (1.115–1.845)	<0.01	1.461 (1.135–1.880)	<0.01
Q3	1.859 (1.466–2.358)	<0.001	1.844 (1.449–2.334)	<0.001	1.844 (1.458–2.332)	<0.001	1.855 (1.465–2.349)	<0.001
Q4	2.914 (2.301–3.689)	<0.001	2.819 (2.198–3.617)	<0.001	3.173 (2.548–3.951)	<0.001	2.966 (2.371–3.712)	<0.001

Model 1: non-adjusted. Model 2: adjusted for age, sex, residence, marital status, education level, household expenditure, alcohol consumption. and nighttime sleep duration. AOR: adjusted odds ratio.

*Smokers*

In univariate analyses, each 1-unit increase in ePWV was associated with a 33.5% increase in CMM risk (HR=1.335; 95% CI: 1.281–1.391) (Table 2). After confounding factors adjustment, the HR for CMM was 1.594 (95% CI: 1.487–1.709). ePWV was divided into quartiles, and participants in the first quartile (Q1) were used as the reference group, with an HR of 1.00. Compared with Q1, the HRs for CMM risk in the Q2, Q3, and Q4 groups were 2.549 (95% CI: 2.001–3.245), 4.112 (95% CI: 3.274–5.166), and 5.735 (95% CI: 4.596–7.158) (all p<0.001). After adjusting for confounding factors, the HR for CMM were 2.797 (95% CI: 2.183–3.584), 4.953 (95% CI: 3.867–6.344), and 8.131 (95% CI: 6.129–10.787) (all p<0.001) in subjects with Q2, Q3, and Q4 compared with those with Q1 values.

**Figure 2:**
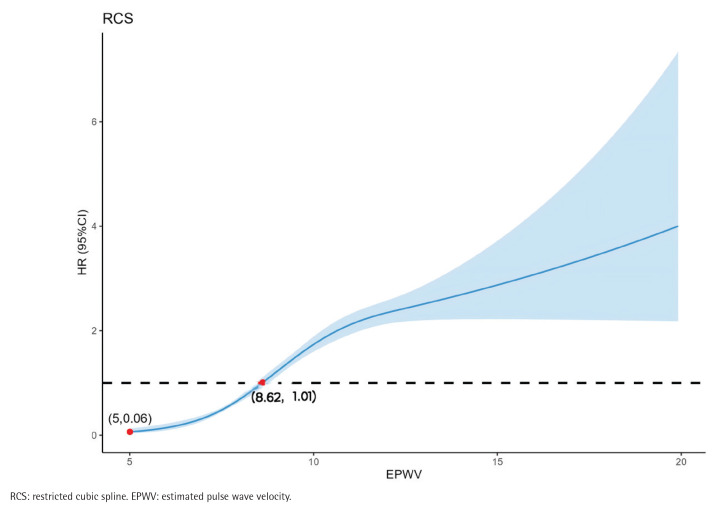
Adjusted restricted cubic spline showing the association between ePWV and incident CMM among CHARLS participants, 2011–2018 (N=8414)

Additionally, the RCS model exhibited a non-linear relationship between ePWV and CMM incidence among all participants (non-linear p<0.001) (Figure 2). As ePWV increased, the HR for CMM gradually increased. The dose–response relationship between ePWV and CMM was consistent with the logistic model.

### Association between WHtR and CMM

*Non-smokers*

In univariate analyses, each 1-unit increase in WHtR was associated with a 108.2% increase in CMM risk (HR=2.082; 95% CI: 1.809–2.396) (Table 2). After confounding factors adjustment, the HR for CMM was 163.107 (95% CI: 61.217–434.583). WHtR was divided into quartiles, and Q1 participants were used as the reference group, with an OR of 1.00. Compared with Q1, the HRs for CMM risk in the Q2, Q3, and Q4 groups were 1.286 (95% CI: 1.011–1.635), 1.859 (95% CI: 1.466–2.358), and 2.914 (95% CI: 2.301–3.689) (all p<0.05). After adjusting for confounding factors, the HR for CMM were 1.27 (95% CI: 1.003–1.624), 1.844 (95% CI: 1.449–2.3334), and 2.819 (95% CI: 2.198–3.617) (all p<0.05) in subjects with Q2, Q3, and Q4 compared with those with Q1 values.

*Smokers*

In univariate analyses, each 1-unit increase in ePWV was associated with a 486.00% increase in CMM risk (HR=5.860; 95% CI: 3.690–9.324) (Table 2). After confounding factors adjustment, the HR for CMM was 236.281 (95% CI: 44.672–1249.744). WHtR was divided into quartiles, and participants in the first quartile (Q1) were used as the reference group, with an HR of 1.00. Compared with Q1, the HRs for CMM risk in the Q2, Q3, and Q4 groups were 1.434 (95% CI: 1.115– 1.845), 1.844 (95% CI: 1.458–2.332), and 3.173 (95% CI: 2.548–3.951) (all p<0.001). After adjusting for confounding factors, the HR for CMM were 1.461 (95% CI: 1.135–1.880), 1.855 (95% CI: 1.465–2.349), and 2.966 (95% CI: 2.371–3.712) (all p<0.001) in subjects with Q2, Q3, and Q4 compared with those with Q1 values.

**Figure 3:**
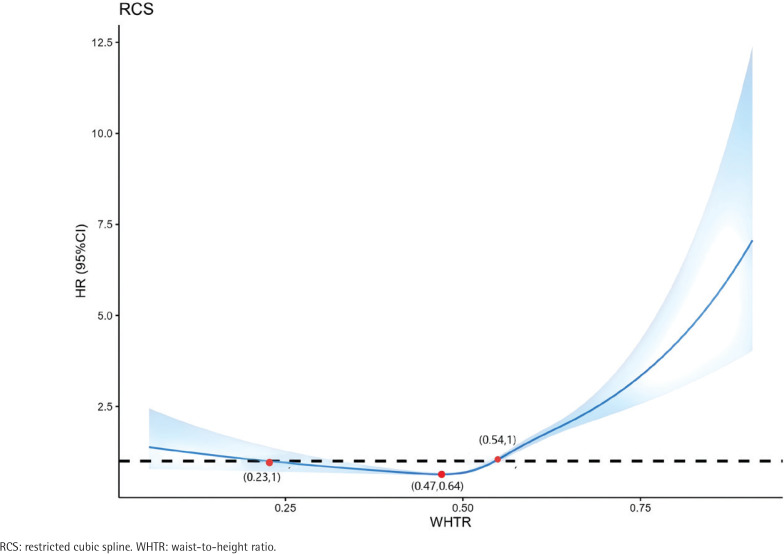
Adjusted cubic spline models showing the association between WHTR and the prevalence of CMM among CHARLS participants, 2011–2018 (N=8414)

The RCS model exhibited a non-linear relationship between WHtR and CMM incidence among all participants (non-linear p<0.001). WHtR and CMM incidence exhibited a U-shaped relationship. When WHtR was >0.47, WHtR positively correlated with CMM incidence, and when WHtR was >0.54, HR was >1 (Figure 3).

### Sensitivity analyses

We conducted several sensitivity analyses to assess the robustness of the interaction between ePWV, WHtR, and CMM episodes. In sensitivity analysis 1, we excluded participants with a history of stroke at baseline (Supplementary file 1). In sensitivity analysis 2, participants with diabetes or who had received diabetes treatment at baseline were excluded (Supplementary file 2). In sensitivity analysis 3, participants with baseline heart disease were excluded (Supplementary file 3). In sensitivity analysis 4, we excluded participants who developed CMM within two years of the baseline survey to minimize latency bias in the results (Supplementary file 4). The results of the sensitivity analyses were consistent with those of this study.

### Mediation analysis

**Figure 4:**
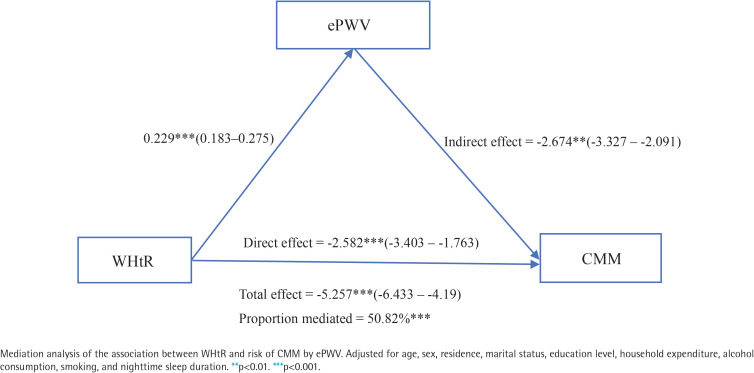
Path diagram of the association between WHtR and CMM, with ePWV as a mediator

Figure 4 illustrates the potential mediating effect of ePWV on the association between WHtR and CMM risk. In the fully adjusted analysis, the ePWV mediation proportion was 50.82% (p<0.001). These results suggest that WHtR may promote CMM development by partially affecting arterial stiffness.

## DISCUSSION

To our knowledge, this is the first large-scale longitudinal study to explore the independent and combined effects of ePWV and WHtR on CMM development in middle-aged and older Chinese adults. Our results indicate that higher ePWV and WHtR are associated with greater CMM. Sensitivity analysis further confirmed these findings. Our results also suggest that WHtR promotes CMM development by partially mediating arterial stiffness effects.

Aortic stiffness is an indicator of subclinical disease and is associated with increased risk of various conditions, including hypertension, CKD, and stroke^16^. ePWV is a new, simple parameter proposed by Greve et al.^17^ to predict aortic stiffness. Studies have demonstrated that ePWV has predictive value for future CVD events, particularly in healthy individuals and untreated hypertensive patients^18,19^. A small-scale study conducted by Ji et al.^20^ in China also revealed that ePWV can predict CVD risk. A longitudinal study conducted by Kim et al.^21^ in South Korea further validated this finding. Our study also found that ePWV was associated with the onset of CMM.

Obesity, particularly abdominal fat accumulation, is closely linked to cardiovascular and metabolic diseases^22^. Previous studies have revealed that obesity is associated with individual CMDs^23^. A study in Iran demonstrated that WHtR can effectively predict CVD occurrence^24^. A study conducted in Medellín, Colombia, similarly found that WHtR is a strong predictor of full cardiometabolic risk (CMR)^25^. A Chinese study demonstrated that WHtR is a potential obesity indicator for distinguishing high CMR, with excellent predictive ability^8^. Another Chinese study exhibited that WHtR can independently predict diabetes risk^26^, consistent with our results.

CMM is defined as the coexistence of two or three CMDs. Given its higher mortality rate and shorter life expectancy, CMM was considered as an outcome measure. The CHARLS database was used to investigate the relationship between WHtR and CMM in middle-aged and older Chinese adults, providing a nationally representative sample. Among the 5996 participants from 2011 to 2018, 1159 (19.33%) had new-onset CMD. Overall, individuals with CMD had lower WHtR and ePWV than those without CMD. Logistic regression analysis illustrated that ePWV and WHtR were independent predictors of CMM. Moreover, RCS analysis adjusted for confounding factors indicated that ePWV was positively correlated with CMM in all participants (non-linear association), whereas WHtR exhibited a U-shaped relationship with CMM incidence. When WHtR exceeded 0.47, it exhibited a positive correlation with CMM incidence.

The exact mechanisms linking obesity, ePWV, and CMM are still unclear. Studies have revealed that as obesity progresses, macrophages infiltrate ectopic fat, and certain adipokines are up-regulated^26^. Adipokines play a crucial role in inflammation pathogenesis and insulin resistance (IR)^26^. As fat tissue accumulates in organs, it disrupts normal metabolic function and ultimately develops systemic metabolic disorders, such as type 2 diabetes and cardiovascular disease^27^.

Our findings indicate a significant association between ePWV, WHtR, and CMM. The mediating role of ePWV between obesity and CMM may result from visceral fat actively secreting excessive inflammatory mediators, which intensify IR and oxidative stress, and worsen endothelial dysfunction^28^, thereby increasing CMM risk. Adipocyte proliferation and hypertrophy may induce adipokine dysregulation^29^, where adipokines are bioactive peptides secreted by visceral adipose tissue with endocrine functions. Adipokine dysregulation can lead to vascular inflammation and remodeling, as well as endothelial dysfunction^30^.

Our study results demonstrated a significant association between smoking and the prevalence of CMM in baseline characteristics, consistent with previous research^4,31^. In the Cox regression analysis, although ePWV and body mass index (WHtR) were independent predictors of CMM in both smoking and non-smoking populations, the hazard ratio (HR) in the smoking group was significantly higher than that in the non-smoking group. This further underscores the importance of smoking cessation as a critical measure for CMM prevention.

This study fills the previous research gap by exploring WHtR and ePWV potential in predicting CMM risk. Our findings remained significant even after adjusting for potential confounding factors. The results remained consistent in sensitivity analyses. Using a large sample from a nationally representative population, we estimated the relationship between WHtR, ePWV, and CMM using Cox regression and RCS models. We also explored the potential mediating role of ePWV in the association between WHtR and CMM risk.

### Limitations

This study has several limitations. First, CMM was determined using self-reported diagnostic information from physicians, without medical record verification, which may have led to classification bias. Second, participants with missing data were excluded during the sample selection process, which may introduce selection bias. Third, the CMM definition used in this study only covers diabetes, heart disease, and stroke. This narrower definition may lead to a potential underestimation of the true prevalence of CMM, thereby affecting the results.

## CONCLUSIONS

Our findings indicate that ePWV significantly mediates the association between WHtR and new-onset CMM, and that both increased ePWV and WHtR are significantly linked with an increased risk of developing CMM. Early identification and intervention of ePWV and WHtR may be useful for CMM prevention and treatment.

## Supplementary Material



## Data Availability

The data supporting this research are available from the following source: The CHARLS dataset is publicly available online, accessible at: http://charls.pku.edu.cn/en
