# Supplementary material for: Estimated pulse wave velocity, waist-to-height ratio, and risk of cardiometabolic multimorbidity: A secondary dataset analysis of the China Health and Retirement Longitudinal Study (CHARLS)
Source: Tob Induc Dis. 2026 Mar 20;24:10.18332/tid/216379. doi: 10.18332/tid/216379 (PMC13005607; doi:10.18332/tid/216379)
Supplement: Supplementary file 1 [file TID-24-40-s1.pdf]

Supplementary Table 1 Hazard ratios and 95 % CI of CMM status for ePWV and WHtR (Participants with baseline stroke were excluded), CHARLS, 2011–2018 (n=8355)

	HR (95 % CI)	P value
**EPWV**	1.513(1.443,1.586)	***
Q1	Ref	
Q2	2.886(2.283,3.648)	***
Q3	5.054(3.989,6.403)	***
Q4	8.869(6.803,11.559)	***
**WHTR**	2.069(1.761,2.433)	***
Q1	Ref	
Q2	1.359(1.118,1.653)	**
Q3	2.003(1.664,2.414)	***
Q4	2.977(2.478,3.578)	***

adjusted for age, sex, residence, marital status, education level, household expenditure, alcohol consumption, smoking and nighttime sleep duration

Abbreviations: WHTR waist-to-height ratio, ePWV estimated pulse wave velocity, HR Hazard ratios

Supplementary Table 2 Hazard ratios and 95 % CI of CMM status for ePWV and WHtR (Participants with baseline diabetes were excluded), CHARLS, 2011–2018 (n=8246)

	HR (95 % CI)	P value
**EPWV**	1.517(1.457,1.592)	***
Q1	Ref	
Q2	3.008(2.410,3.947)	***
Q3	5.483(4.176,6.998)	***
Q4	9.658(7.271,12.589)	***
**WHTR**	2.056(1.714,2.422)	***
Q1	Ref	
Q2	1.367(1.101,1.614)	**
Q3	2.018(1.662,2.438)	***
Q4	3.004(2.483,3.621)	***

adjusted for age, sex, residence, marital status, education level, household expenditure, alcohol consumption, smoking and nighttime sleep duration
Abbreviations: WHTR waist-to-height ratio, ePWV estimated pulse wave velocity, HR Hazard ratios

Supplementary Table 3 Hazard ratios and 95 % CI of CMM status for ePWV and WHtR (Participants with baseline heart disease were excluded), CHARLS, 2011–2018 (n=8052)

	HR (95 % CI)	P value
**EPWV**	1.512(1.435,1.576)	***
Q1	Ref	
Q2	3.028(2.385,3.912)	***
Q3	5.502(4.278,6.955)	***
Q4	9.613(7.285,12.617)	***
**WHTR**	2.055(1.754,2.429)	***
Q1	Ref	
Q2	1.333(1.101,1.631)	**
Q3	1.978(1.512,2.322)	***
Q4	2.997(2.412,3.578)	***

adjusted for age, sex, residence, marital status, education level, household expenditure, alcohol consumption, smoking and nighttime sleep duration
Abbreviations: WHTR waist-to-height ratio, ePWV estimated pulse wave velocity, HR Hazard ratios

Supplementary Table 4 Hazard ratios and 95 % CI of CMM status for ePWV and WHtR (Participants who developed CMM within two years were excluded), CHARLS, 2011–2018 (n=8307)

	HR (95 % CI)	P value
**EPWV**	1.571(1.489,1.677)	***
Q1	Ref	
Q2	3.112(2.475,4.010)	***
Q3	5.575(4.356,7.152)	***
Q4	9.687(7.341,12.751)	***
WHTR	2.102(1.792,2.488	***
Q1	Ref	
Q2	1.383(1.135,1.702)	**
Q3	2.039(1.687,2.464)	***
Q4	3.014(2.501,3.647)	***

adjusted for age, sex, residence, marital status, education level, household expenditure, alcohol consumption, smoking and nighttime sleep duration
Abbreviations: WHTR waist-to-height ratio, ePWV estimated pulse wave velocity, HR Hazard ratios

© 2026 Fei Z. et al.
